# Among the Boards of Examiners

**Published:** 1900-09

**Authors:** W. W. Thorburn

**Affiliations:** Secretary, Lansing, Mich.


### AMONG THE BOARDS OF EXAMINERS.

Michigan State Veterinary Board. This Board met at the Hotel Butler, Lansing, August 7th, for its regular semi-annual meeting for the examination of non-graduates for registration, and also the examination of affidavits of graduates. There were in all 120 applications before the Board: 90 of which were graduates, representing nine different schools, and 30 non-graduates who had to take the examination, 3 of whom passed. The subjects on which they were examined were: Anatomy, Pathology, Physiology, and Materia Medica. When the Board were making out certificates they were compelled to refuse granting the documents to certain applicants, otherwise meeting the provisions of the law, because they have not yet been naturalized; the fact that they have declared their intentions of becoming citizens makes them aliens notwithstanding. After declaring his intentions, the alien must remain in this country two years before being naturalized. Naturalization papers cannot be granted unless the alien has been a resident of this country for five years previously. The Secretary of the Board has authority from the Attorney-General to refuse granting certificates to those who in their applications specified that they had merely declared their intentions of becoming citizens. Graduates of the Ontario Veterinary College, since January 1, 1900, are required to pass an examination before the Board.

W. W. Thorburn,
Secretary, Lansing, Mich.

New York State Veterinary Board.—The following eleven have passed the examination and received the license of this Board from September, 1899, to June, 1900, inclusive: Drs. Francis C. Edmonds, Charles B. Potter, W. Lincoln Bell, Adolph Eichorn, Daniel J. Mangan, Charles N. Jewell, John W. Corrigan, Clarence L. Barnes, Grant S. Hopkins, William J. Mitchell, and Louis Juliand, of New York State, and representing the New York College of Veterinary Surgeons, New York State Veterinary College, McGill University, Veterinary Department, and New York American Veterinary College.

